# Curcumin micelles entrapped in eudragit S-100 matrix: a synergistic strategy for enhanced oral delivery

**DOI:** 10.2144/fsoa-2020-0131

**Published:** 2021-01-18

**Authors:** Helmy Yusuf, Rizka Arifa Rahmawati, M Agus Syamsur Rijal, Dewi Isadiartuti

**Affiliations:** 1 Department of Pharmaceutics, Faculty of Pharmacy, Universitas Airlangga, Jl Mulyorejo Surabaya 60115, Indonesia

**Keywords:** curcumin, eudragit s-100, micelles, poloxamer 407, powder, spray drying

## Abstract

**Background:**

Therapeutic activities of curcumin (CUR) via oral administration are hampered by the lack of bioavailability due to its poor water solubility and rapid degradation in GI tract.

**Materials & methods:**

This preliminary study developed CUR micelle-eudragit S100 (EUD) dry powder (CM-EDP) spray-dried formulations. Poloxamer 407 was used as a micelle-forming agent and EUD as an entrapping matrix for protection over hydrolysis and enzymes in the GI tract.

**Results:**

The morphology of CM-EDP showed agglomeration with cratering on the surface of particles. Differential thermal analysis and x-ray diffractometry data exhibited evidence that CUR was converted into amorphous solid. An increased concentration of micelle-forming and dispersion matrix polymers resulted in a high fraction of drug being converted into the amorphous state. A significant increase in dissolution by 7–10 times was achieved compared with that of raw CUR.

**Conclusion:**

The present study disclosed the CM-EDP potency for future development of CUR oral formulation.

## Lay abstract

Curcumin (CUR) is a natural compound that shows several pharmacological activities, including anti-inflammatory and potential actions against Parkinson’s and Alzheimer’s. However, several drawbacks need to be addressed its application as a therapeutic agent via oral administration. These drawbacks include its poor water solubility and rapid degradation in the GI tract. The present study developed CUR micelle-eudragit S100 (EUD) dry powder formulation involving poloxamer 407 as solubilizing agent and EUD as entrapping matrix for protection in acidic environments and the enzymes in the GI tract. The final product is in the form of dry powder, which showed potency in enhancing CUR absorption following oral administration.

Curcumin (CUR) is a hydrophobic polyphenol derived from *Curcuma longa* with several potential therapeutic activities, including in cancer, Alzheimer’s, Parkinson’s and cardiovascular disease [1–3]. Oral administration of CUR is hampered by its low bioavailability and absorption in the GI tract owing to its poor water solubility [4,5].

A well-designed delivery system that allows CUR to access the target site at sufficient concentration is required. In addition, given that oral administration is still the most popular and acceptable route compared with other nonparenteral routes, the development of a CUR oral formulation has gained wide interest [6,7]. The main challenges in the development of a CUR oral formulation are the lack of aqueous solubility and rapid degradation caused by hydrolysis and rapid metabolism by enzymes in the GI tract. Various approaches, including the use of carriers such as solid lipid nanoparticles, liposomes, macroemulsions and polymeric micelles, have been implemented [8–10]. Encapsulation in spray-dried microparticles using whey protein as the matrix has also been used to increase CUR absorption [11]. Furthermore, a comprehensive review of CUR development to enhance its bioavailability in various solid forms has been performed [12].

In addressing the drug solubility problem, the use of polymeric micelles is of particular interest. Micelles are composed of polymeric molecules that assemble themselves into a spherical structure in aqueous solutions, driven by their amphiphilic nature [13,14]. The increased solubility of hydrophobic drugs through micellization occurs due to drug entrapment in the nonpolar core, where they are surrounded by polar groups [15]. Micelles increase drug absorption and bioavailability in the GI tract [16,17]. Poloxamer 407 (POL) is one of the well-known micelle-forming polymers that have gained considerable attention in the last decades [18–20]. This compound is a synthetic polymer that consists of block copolymers of ethylene oxide and propylene oxide [17,21].

Other considerations in the development of CUR oral formulations are the low gastric absorption, rapid metabolism by enzymes and absorption in the intestine at pH 6.8–7 [22–24]. Therefore, a polymer that carries CUR for release in the intestine would be advantageous. Such properties are perfectly fulfilled by eudragit S100 (EUD), a synthetic polymer with pH-dependent solubility [25–27]. EUD is composed of methacrylic acid and methyl methacrylate, which are soluble in neutral to an alkaline environment [28,29].

In this preliminary study, the entrapment of CUR micelles into a polymeric matrix in the form of dry powder was carried out. The developed CUR micelle-EUD dry powder (CM-EDP) formulations were expected to enhance the solubility, offer protection against GI tract environment and facilitate drug release in the intestine where the pH is neutral to weakly alkaline. These features can offer new strategies for the improvement of CUR bioavailability upon oral administration. The physical properties and characteristics of CUR micelles-dry powder were investigated using scanning electron microscopy (SEM), differential thermal analysis (DTA) and x-ray diffractometry (XRD). The dissolution profile of the developed CM-EDP formulations was also investigated.

## Materials & methods

### Materials

CUR, POL and phosphate buffer were purchased from Sigma Aldrich (Singapore). EUD was purchased from Evonik Industry (Indonesia). Ethanol was purchased from E-Merck (Indonesia).

### Preparation of CM-EDP

CUR in ethanol solution was added dropwise to 50 ml POL aqueous solution under constant stirring at 800 rpm. A weighed amount of EUD was completely dissolved in 200 ml phosphate buffer (pH 7) and further added to the micellar solution of CUR and POL under a constant stirring at 700 rpm for 4 h. The final mixture was spray dried (Buchi B-290 Mini-Spray Dryer, Flawil, Switzerland) to obtain a dry powder. The spray dryer condition settings were as follows: inlet/outlet temperatures of 150/90°C, air flow rate of 320 l/h, aspiration air of 90%, feed flow of 5 ml/min and spraying pressure of 5.0–5.8 mbar. Dry products of CM-EDP were stored in a tight container for further analysis. Four different formulations of CM-EDP were prepared to achieve the following molar ratios of CUR:POL:EUD: 1:0.6:1.3 (F1), 1:0.7:1.4 (F2), 1:0.8:1.5 (F3) and 1:0.9:1.6 (F4). A physical mixture (PM) was prepared using the same ratio as F1, which contained the lowest amount of polymer.

### Determination of powder yield

Powder yield was determined by the percent weight ratio between the total weight of recovered powder and the total weight of the initial solid mass fed to the spray dryer. The powder yield percentage was obtained by the following equation:

% Powder yield = (total weight of collected powder)/(total weight of initial solid) × 100%

### Scanning electron microscopy

The morphology of CM-EDP was analyzed using SEM (Phenom, OR, USA). Prior to examination, dry powder samples were scattered on a carbon tip and sputter coated with Au-Pd alloy followed by vacuuming for 30 min. The samples were further examined under the microscope.

### Differential thermal analysis

A Mettler Toledo FP85 TA Cell DTA (Switzerland) was used for recording the DTA thermograms of all samples, including the raw materials, CM-EDP formulations prepared by spray drying and the PM. The samples (3–5 mg) were heated in a closed aluminium crimped pan at a rate of 20°C/min, within the temperature range of 50–300°C.

### X-ray diffractometry

X-ray powder diffraction patterns were analyzed using a Philips X′Pert PRO (Panalytical, The Netherlands) x-ray diffractometer using a voltage of 40 kV and a 30 mA current. The scanning rate employed was 2°/min over the 5–50° 2θ range. The XRD patterns of all raw materials, CM-EDP formulations prepared by spray drying and the PM were recorded.

### Dissolution study

CM-EDP powder was made into a mini tablet containing 1.0 mg CUR by using a single-punch hydraulic press (Natoli Engineering Company, MO, USA) with a force of 2000 N. Dissolution studies were performed using 500 ml dissolution medium (phosphate buffer pH 6.8) at temperature of 37 ± 0.5°C and under constant stirring at 100 rpm for 30 min. Tablets of CM-EDP formulations were added to the dissolution medium. Dissolved CUR was sampled at fixed time intervals (5, 10, 15, 20, 25 and 30 min) and assayed by measuring the absorbance at 421 nm using a high-liquid performance chromatography (Agilent 1100 series, Agilent Technologies, Waldbronn, Germany). Every 5.0 ml of each sampled volume was replaced with equal volume of fresh dissolution medium to keep the volume constant during the test.

### Statistical analysis

Statistical analysis was performed by use of one-way analysis of variance to assess the significance of the difference among mean values. Statistical probability (p) values less than 0.05 were considered significantly different.

## Results & discussion

### Powder yield (%)

**Figure 1.**
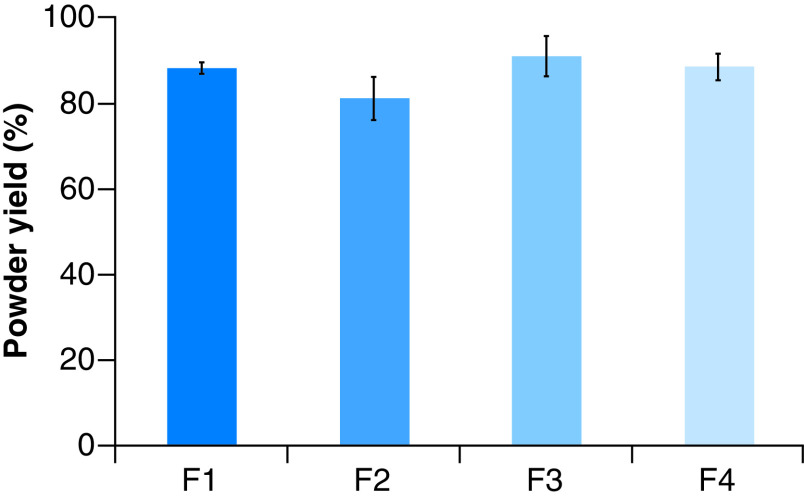
Powder yield (%) of curcumin micelle-EUD dry powder formulations prepared at different molar ratios of curcumin:poloxamer407:eudragit S100. (F1) 1:0.6: 1.3; (F2) 1:0.7:1.4; (F3) 1:0.8:1.5 and (F4) 1:0.9:1.6.

The powder yield (%) was calculated based on the percentage of the recovered solid mass after spray drying, relative to the weight of solid mass contained in the liquid feed. The recovered powder at the bottom of the cyclone was weighted shortly after spray drying. The powder yield of all CM-EDP formulations were as follows: 88.0 ± 1.4% (F1), 81.0 ± 5.0% (F2), 90.7 ± 4.7% (F3) and 88.2 ± 3.1% (F4) (Figure 1). The powder yield of the CM-EDP formulations varied regardless of the increased amount of polymer used in the formulations. All developed CM-EDP formulations showed yields in the range of 80–90%. No significant difference (p > 0.05) was observed in the powder yield among CM-EDP formulations.

The use of polymer, in other words, EUD, in the developed CM-EDP formulations showed a positive effect on powder yield, and this result can be attributed to the increased solid mass of the dried particles. In addition, EUD decreased the stickiness of fine particles to the drying chamber wall. In the present study, EUD was used as a dispersion matrix for CUR micelles and to increase the bulk density, successfully improving the drying yield (Figure 1). Using a high amount of polymer improved the drying yield due to the increased solid mass of the final dried powder [30,31]. Nevertheless, a high polymer concentration may also increase the viscosity of feed solution, which causes a slow spray rate and further decreases the final powder yield [32].

### Morphology

**Figure 2.**
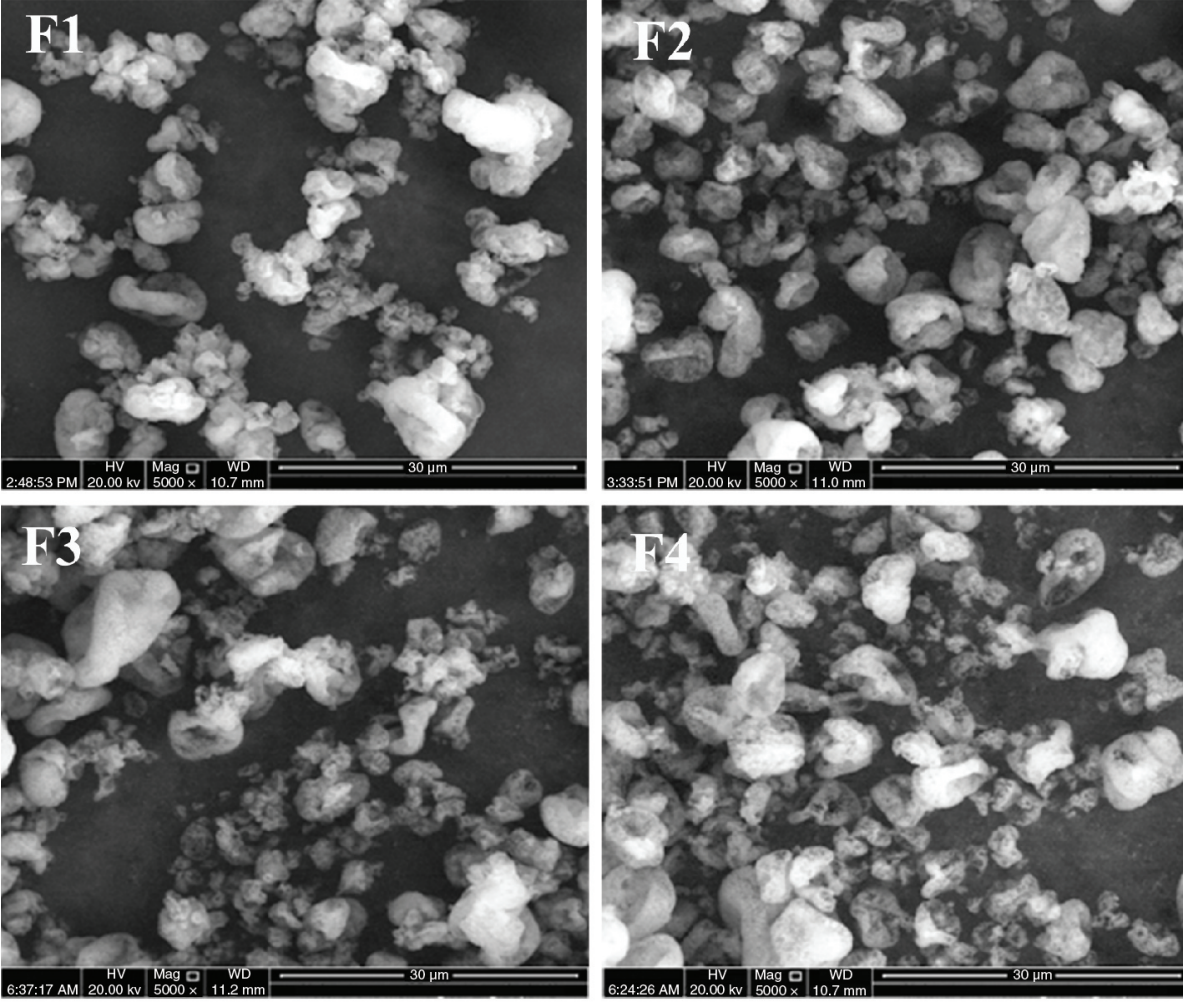
Scanning electron microscopy images of curcumin micelle-eudragit s-100 dry powder formulations prepared at different molar ratios of curcumin:poloxamer 407:eudragit S100. (F1) 1:0.6: 1.3; (F2) 1:0.7:1.4; (F3) 1:0.8:1.5 and (F4) 1:0.9:1.6.

Spray drying produced relatively similar morphology of CM-EDP powders for all formulations. SEM results revealed that the particles varied in shape and size. Their morphology exhibited an agglomerate with cratering on the surface of particles (Figure 2). Such agglomeration might have been facilitated by the formation of a solid bridge, as indicated by a typical structure of polymeric appearance. The high temperature used during spray drying might have caused the EUD to undergo phase transition, creating a continuous solid phase that bound the smaller particles.

Atomization in the spray-drying condition was set to produce droplets with a size of approximately 10 μm in diameter. The droplets might have shrunk extremely upon contact with hot air. SEM results showed that most particles varied in shape and size (Figure 2). Large and irregular particles with a size up to 10 μm and those with a more regular structure and smaller size up to 1 μm in diameter were captured in the SEM images. The structural plasticity of the particles during drying might have contributed to the irregularities of particle surface morphology [33]. Furthermore, their physical morphology showed an agglomerate-like appearance with cratering on the surface structures. To a certain extent, such agglomerate particles might have been facilitated by polymeric solid bridges, in which the typical structure of polymeric appearance was evident [34–36]. As the water evaporated, the EUD remained and formed a continuous solid phase that bound the small particles [37,38]. Moreover, this might have been caused by the permeable nature of the EUD, which allowed the movement of water vapor from the interior to the surface of particles. As the internal pressure decreased, water vapor diffused through the particle structure, resulting in an inward collapse that formed the crater.

### Thermal properties

**Figure 3.**
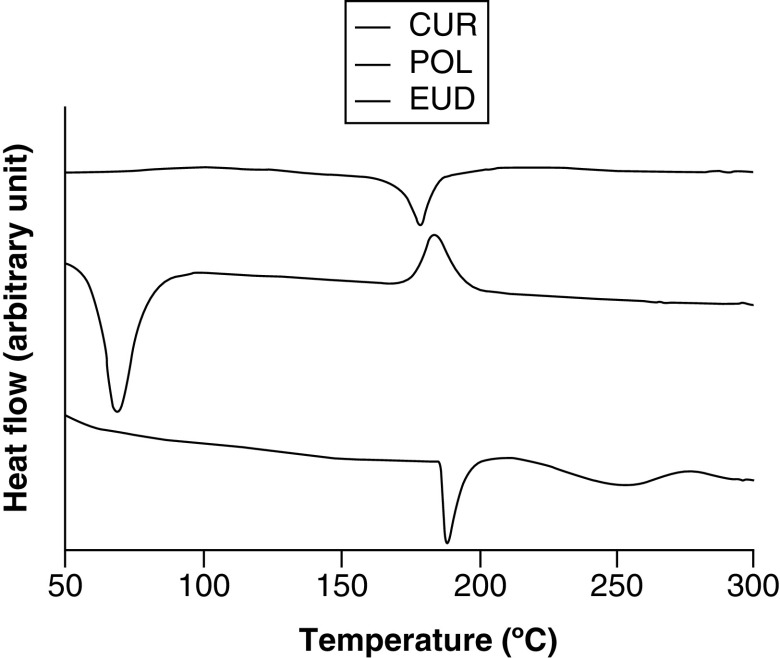
Differential thermal analysis thermograms of CUR, EUD, POL. CUR: Curcumin; EUD: eudragit s-100; POL: Poloxamer 407.

Figure 3 shows the thermograms of crude components. CUR is a crystalline material, as shown by its sharp endothermic peak at 178°C. EUD demonstrated two endothermic peaks. The first sharp peak was at 189°C, indicating an ordered-like structure, whereas the second peak was notably broader at 250°C. POL showed an endothermic peak at 69°C and an exothermic peak at 187°C, indicating that the material was crystalised upon heating.

**Figure 4.**
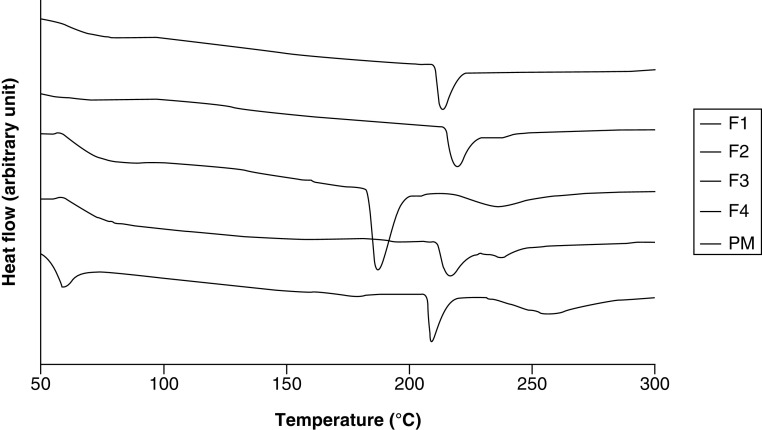
Differential thermal analysis thermograms of curcumin micelle-eudragit s-100 dry powder formulations prepared at different molar ratios of curcumin:poloxamer 407:eudragit S100. (F1) 1:0.6: 1.3; (F2) 1:0.7:1.4; (F3) 1:0.8:1.5 and (F4) 1:0.9:1.6. Their PM was prepared using the same ratio as F1. PM: Physical mixture.

Figure 4 presents the thermograms of all CM-EDP formulations together with their PM. The thermogram of PM indicated phase separation of every single component in the mixture. Three peaks were observed at 60, 200 and 250°C, corresponding to POL, CUR and EUD, respectively. By contrast, the endothermic peaks attributed to CUR were not observed in three CM-EDP formulations (F1, F2 and F4). F1 and F2 showed a single endothermic peak at high temperatures around 215 and 220°C, respectively. F4 showed two peaks at 218°C and a small peak at 238°C, indicating that two populations occurred in the sample.

The DTA data showed interesting results (Figures 3 & 4). The endothermic peaks attributed to CUR were not observed in three CM-EDP formulations (F1, F2 and F4). F1 and F2 showed a single endothermic peak, whereas F4 showed two peaks, indicating that two populations existed in this formulation. A different result was shown by F3, whose recognizable peak of CUR was still observed. The disappearance of CUR peaks in F1, F2 and F4 indicated the miscibility among components and transformation of CUR into an amorphous state [39,40]. The CUR might have been homogeneously distributed in the polymeric matrix. Nevertheless, the strength of their intermolecular bonds resulted in a typical endothermic peak for an ordered structure material [41]. In the case of F4, the two populations possibly included two CUR regions, in other words, CUR-rich and CUR-poor regions. F3 presented a different result, in other words, a single endothermic peak at 188°C. The existing peak can be attributed to CUR molecules which might have been phase separated from the mixture [42,43]. This anomalous result can be caused by the increased concentration of polymers in the mixture, resulting in a concentrated CUR that was difficult to distribute.

### Crystallinity

**Figure 5.**
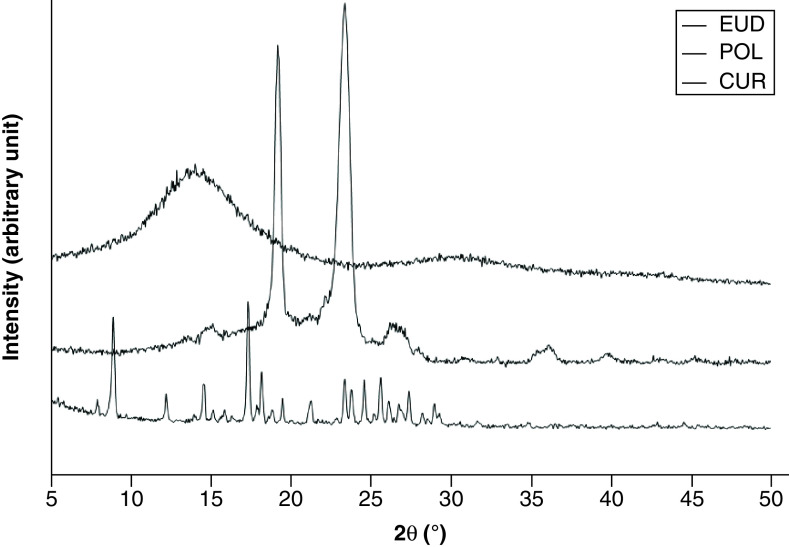
Powder x-ray diffractograms of CUR, EUD, POL. CUR: Curcumin; EUD: eudragit s-100; POL: Poloxamer 407.

Figure 5 presents the powder x-ray diffractograms of the raw materials. CUR showed intensive and sharp peaks, whereas POL showed less intensive peaks, implying that these materials existed as an ordered crystalline material. EUD is an amorphous powder without crystalline structure, as suggested by the observed diffractogram.

**Figure 6.**
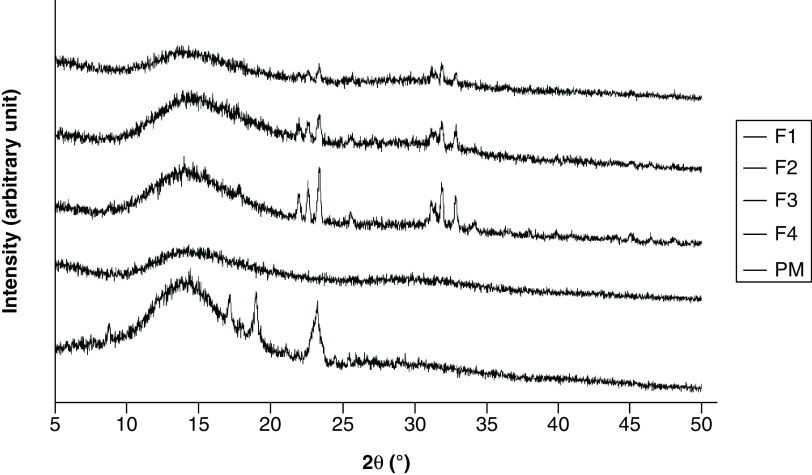
Powder x-ray diffractograms of curcumin micelle-eudragit s-100 dry powder formulations prepared at different molar ratios of curcumin:poloxamer407:eudragit S100 and their physical mixture. (F1) 1:0.6:1.3; (F2) 1:0.7:1.4; (F3) 1:0.8:1.5 and (F4) 1:0.9:1.6. Their PM was prepared using the same ratio as F1. PM: Physical mixture.

Figure 6 shows the diffractograms of PM and CM-EDP formulations. The PM exhibited several sharp peaks, implying the presence of crystalline solid attributable to CUR masked by the polymers. The sharp and intensive peaks were extremely reduced in the F1, F2 and F3 of CM-EDP formulations. Nevertheless, small and recognizable peaks remained in the three formulations, indicating that a small part of CUR was possibly retained as crystalline.

Figure 5 represents XRD data of raw materials, whereas Figure 6 provides the CM-EDP formulations. Typical sharp and intensive peaks attributed to CUR in the x-ray diffractogram were extremely reduced in the F1, F2 and F3 of CM-EDP formulations (Figure 6). Nevertheless, several small recognizable peaks remained, indicating that a small portion of CUR might still be present in crystalline state. Therefore, the CUR might have existed as partially crystalline and partially amorphous in the three formulations [5,44]. Interestingly, these small peaks became more evident as the amount of EUD increased. F3 showed the highest intensity compared with F1 and F2, which have been identified as phase separated by the DTA results (Figure 4). Thus, the amount of EUD up to this level (F1–F3) was insufficient to inhibit the recrystallization of CUR during the drying process [39,45]. F4 was the only formulation that indicated an amorphous solid in which CUR was totally transformed to an amorphous state. Given that F4 contained the highest amount of EUD, this result confirms that the degree of amorphization is a function of EUD concentration. This finding was also supported by DTA data, which revealed that F4 possessed characteristics of a disordered structure (Figure 4).

### Dissolution study

**Figure 7.**
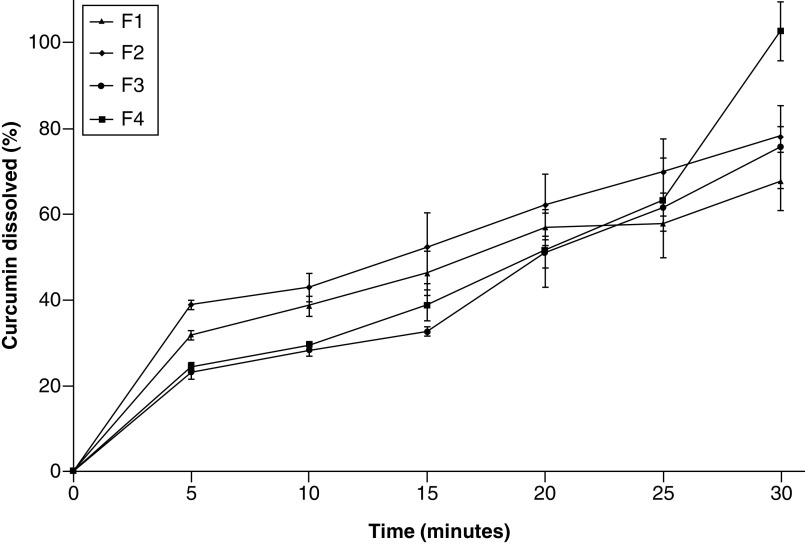
*In vitro* curcumin release (%) from curcumin micelle-eudragit s-100 dry powder formulations prepared in phosphate-buffered saline (pH 6.8) at 37°C at different molar ratios of curcumin:poloxamer 407:eudragit S100. (F1) 1:0.6:1.3; (F2) 1:0.7:1.4; (F3) 1:0.8:1.5 and (F4) 1:0.9:1.6.

The polymer used in this study is pH sensitive in terms of its nondissolution in acidic environments. Thus, the drug will not be released or may be released in a negligible amount in acidic medium. Therefore, the study focused on the release properties in the intestine with pH 6.8. Figures 7 show the dissolution curves of CM-EDP formulations. The results showed that the total dissolved CUR from all the developed CM-EDP formulations at the end of experiment were 67.80 ± 6.80%, 78.22 ± 2.37%, 75.79 ± 9.73% and 102.80 ± 6.92% for F1, F2, F3 and F4, respectively. Based on the XRD results, all formulations were partially crystalline, in which the solubility was determined by the saturation concentration of the amorphous part at the surface of particles. Therefore, in semicrystalline systems, the solubility is influenced by the amount and structure of solid state on the particle surfaces that are in equilibrium contact with the solution.

The release profiles of all formulations were relatively similar in the earlier period. However, F4 showed a sudden increase in the last minutes of observation. No significant difference (p > 0.05) was observed in the percentage of dissolved CUR among CM-EDP formulations. Drug release from F4 at the last minutes might have occurred through the remaining fragments of the matrix which was eroded by intestinal pH at prolonged periods.

The dissolution profiles of all developed CM-EDP formulations showed a percentage of dissolved CUR in the predetermined time of experiment (Figure 7). All CM-EDP formulations showed a linear increase with the increase in time. Only F4 experienced a burst increase in the last minutes. No significant difference (p > 0.05) was recorded in the percentage of dissolved CUR among CM-EDP formulations. The sudden increase in F4 can be due to the nature of EUD, that is, the burst release occurred through the remaining fragments of the matrix, which was eroded at prolonged periods. The solubility of raw CUR must be considered to explain the effect of CM-EDP formulations on the improved CUR dissolution. Raw CUR is insoluble in acidic to neutral pH, and similar studies have shown that the dissolved raw CUR was negligible [46,47]. A study reported the dissolved raw CUR was 0.2 μg/ml following a 60 min experiment; the value equaled 100 μg in 500 ml medium [48]. In the present study, the developed CM-EDP formulations contained 1000 μg CUR with the percentage of dissolved CUR in the range of 67.8–102.8%; the amount ranged from 678 to 1000 μg in 500 ml medium. This finding means that the enhanced dissolution of CUR loaded in CM-EDP formulations increased by approximately 7–10 times than that of the raw CUR. In this regard, the application of micelle-forming polymer (POL) and dispersion matrix polymer (EUD) in the CM-EDP formulation might have been beneficial to overcome the dissolution problem. Drug dissolution can be expressed by various techniques using derived dissolution parameters to explain details of the dissolution properties of the tested formulations [49]. However, in the present study, drug dissolution was expressed as the quantity of drug dissolved at a specified time and provided as a cumulative percentage of the drug dissolved at the end of the experiment. Given that the raw CUR only showed negligible release, the increased dissolution was a function of the developed formulation.

Several mechanisms were proposed to explain the enhanced dissolution of CUR from the developed formulations. These mechanisms include the reduced crystal size of CUR, solubilization effect of POL through micellization and conversion of CUR to amorphous solid, as confirmed by DTA and XRD data. POL enhanced the drug solubility through micellization mechanism, whereas EUD protected the entrapped CUR from hydrolysis and enzymes in the GI tract. EUD also enhanced the drug release at the designated pH (6.8). The level of dissolved CUR increased with the increase in the proportion of molar ratio between POL and EUD. The optimum molar ratio of CUR:POL:EUD was approximately 1:0.9:1.6 as observed in F4. The high polymer concentration used in the system was a determining factor for the improved dissolution process of the CM-EDP formulation.

## Conclusion

In this preliminary study, the CM-EDP formulations were developed and investigated in terms of their physical characteristics and dissolution profiles. Different drug and polymer ratios were employed. High concentrations of micelle-forming polymer and the dispersion matrix polymer resulted in a high fraction of drug converted into amorphous state. This phenomenon led to an increased percentage of the dissolved drug. From the physical characteristics and dissolution studies of the developed formulations, F4 was the best formulation. Furthermore, the results suggest that POL and EUD synergistically improved the dissolution of CUR formulated in CM-EDP, which may further improve the absorption over oral administration. This study provides evidence for future development of CUR oral delivery in the form of CM-EDP formulation.

## Future perspective

Oral bioavailability is one of the challenging issues in formulation development of active pharmaceutical ingredients classified as biopharmaceutical Class II such as CUR. The fundamental problem is related to poor aqueous solubility. On the other hand, enhancement of bioavailability following oral administration can improve the therapeutic effectiveness of active pharmaceutical ingredients. Thus, enhancing CUR solubility in aqueous environment is the main goal in the development of solid dispersion-based formulation. The use of high dosage of water-insoluble drugs in conventional formulation has been an issue due to the ineffectiveness of dosage in the treatment. Given this condition, CM-EDP formulations were developed by employing POL as the micelle-forming polymer and EUD as a pH-sensitive matrix. Spray-drying technique was used to obtain the dry product. The present study revealed that incorporating CUR in POL is advantageous because it helped in the transformation of CUR from crystalline to amorphous state through micellar solubilization mechanism. EUD is also beneficial for the protection of CUR against hydrolysis and enzymes in the GI tract. The dissolution study of CM-EDP showed an enhanced dissolution rate >80% at which CUR was dissolved within 30 min. Based on this finding, the future development of CM-EDP in the form of sublingual tablets may provide an effective dosage form for CUR oral administration. Further investigations are required to study the performances of the developed sublingual tablet. Several approaches in the development of sublingual tablet can be the following; investigation of tablet physical characteristics, including hardness, friability and disintegration time; exploration of techniques in terms of tablet manufacturing aspects. Another important investigation will be in the aspect of physicochemical stability during storage, which is advantageous for complementary findings. We have conducted the stability test for the dry product in a wide range of pH and temperatures. However, robust techniques and methodology are required to ensure consistent results. In addition, efficiency studies for other drying techniques can provide additional insights into the manufacture of solid dispersion powder. This aim will be included in future studies to assess the effectiveness of selected manufacturing procedures. Key indicators can still be addressed considering the solid state of drugs in the dispersion matrix.

**Summary points**

- The development of CUR micelle-EUD dry powder (CM-EDP) spray-dried formulations for oral delivery has been described in this manuscript.
- The CM-EDP spray-dried formulations were formulated using poloxamer 407 (POL), a synthetic micelle-forming polymer and further entrapped in the eudragit S100 (EUD) matrix.
- This combination strategy is significant to overcome the bioavailability problems through several mechanisms, including: enhancing drug solubility; protecting curcumin (CUR) against hydrolysis and enzymes in the GI tract and; facilitating drug release in the intestine where the pH is neutral to weakly alkaline.
- The solubilizing effect of POL may be achieved through micellisation and the conversion of CUR to amorphous solid as confirmed by differential thermal analysis and x-ray diffractometry data.
- EUD offered protection to the entrapped CUR from hydrolysis and enzymes in the GI tract and enhanced the drug release at the designated pH (6.8).
- Broad phase transition of CUR in the form of CM-EDP, as observed in differential thermal analysis thermogram, and the disappearance of peaks attributed to the CUR crystals shown by the XRD diffractogram indicate that the CM-EDPs were amorphous.
- The dissolved CUR from CM-EDP formulations showed that the use of POL enhanced the dissolution rate of CUR.
- CM-EDP offers opportunity to the future development of effective formulations that improve CUR bioavailability for oral administration.
